# Perioperative intravenous lidocaine infusion for postsurgical pain management in bariatric surgery patients

**DOI:** 10.1186/s44158-024-00208-9

**Published:** 2024-10-30

**Authors:** Gilberto Duarte-Medrano, Natalia Nuño-Lámbarri, Analucia Dominguez-Franco, Yazmin Lopez-Rodriguez, Marissa Minutti-Palacios, Adrian Palacios-Chavarria, Luigi La Via, Daniele Salvatore Paternò, Giovanni Misseri, Giuseppe Cuttone, Massimiliano Sorbello, Guillermo Dominguez-Cherit, Diego Escarramán

**Affiliations:** 1 Anesthesiology Department of Medica Sur Clinic & Foundation, Mexico City, Mexico; 2 Anesthesiology Department of the ABC Campus Observatorio Medical Center, Mexico City, Mexico; 3 Traslational Research Unit, Medica Sur Clinic & Foundation, Puente de Piedra 150, Toriello Guerra, Mexico City, Tlalpan 14050 Mexico; 4 Department of Surgery, Faculty of Medicine, The National Autonomous University of Mexico (UNAM), Copilco Universidad, Escolar 411A, Mexico City, Coyoacán Mexico; 5 Intensive Care Department of the ABC Campus Observatorio Medical Center, Mexico City, Mexico; 6 Department of Anesthesia and Intensive Care, University Hospital Policlinico “G. Rodolico – San Marco”, Via Santa Sofia, 78, Catania, 95123 Italy; 7 Department of Anesthesia and Intensive Care, Hospital “Giovanni Paolo II,” ASP Ragusa, Ragusa, Italy; 8 Fondazione Istituto “G. Giglio” Cefalù, Palermo, Italy; 9 Department of Anesthesia and Intensive Care, ASP Trapani, Trapani, Italy; 10 Faculty of Medicine and Surgery, University “Kore, Enna, Italy; 11 Escuela de Medicina, Tecnológico de Monterrey, Mexico City, Mexico; 12 Anesthesiology Department of Medical Center Sigle XXI, Mexico City, Mexico

**Keywords:** Analgesia, Bariatric surgery, Enhanced recovery after surgery, Nausea, Post operative nausea and vomit

## Abstract

**Introduction:**

Obesity is one of the biggest modern health issues worldwide. Owing to the failure of both behavioral and pharmacological measures, the surgical approach has been established as the main conduct to follow, with bariatric surgery being one of the most effective and safe procedures. One of the bases for the optimal analgesic strategy is the use of adjuvants during the perioperative period. One of the main drugs in use is lidocaine.

**Aim:**

To evaluate postoperative pain after perioperative lidocaine infusion in patients undergoing bariatric surgery and describe the presence of nausea and vomiting during the first 24 h.

**Methods:**

This was a retrospective study of patients who underwent laparoscopic bariatric surgery at ABC Medical Center. Two study arms were established: a group of patients who received lidocaine infusion and a control group. The presence of pain, nausea, or vomiting was evaluated upon admission to the recovery area and 1 h and 24 h after the intervention. The normal distribution of the data was first verified via the Shapiro–Wilk test. The data are presented as medians for quantitative variables and as frequencies for qualitative variables.

**Results:**

A total of 50 surgeries were performed, with a significant correlation between lidocaine infusion and lower pain values at 1 h (*p* = 0.04). Similarly, there was a marked trend in the presence of nausea in control group 4 (18.6%) vs. 15 (53.5%).

**Conclusions:**

Our data suggest that the use of intraoperative lidocaine infusion is limited in postoperative pain management; nonetheless, it significantly improves the incidence of postoperative nausea.

## Introduction

Obesity is one of the most pressing global health challenges and is defined by the World Health Organization (WHO) as a body mass index (BMI [kg/m^2^]) exceeding or equal to 30 [1]. The current projections suggest that 39–49% of the world’s population is either overweight or obese. This rise in obesity rates has been observed universally across both sexes, with a notable propensity toward higher prevalence among females [2, 3]. In Mexico specifically, nearly 40% of the adult population is affected by obesity, with forecasts indicating a 54% increase in obese men and a 37% increase in obese women by the year 2050. Notably, there is a discernible trend wherein the prevalence of obesity is expected to surpass that of overweight [4]. In light of the therapeutic shortcomings of conventional behavioral and pharmacological interventions, surgical interventions have emerged as primary modalities, with bariatric surgery recognized as one of the most efficacious and safe contemporary procedures[5, 6]. The updated Enhanced Recovery After Surgery (ERAS) protocol for bariatric surgery, published in 2022, underscores the importance of employing short-acting drugs and reducing opioid consumption as fundamental tenets of perioperative management [7]. Among the pharmacological agents utilized, lidocaine—a local anesthetic belonging to the amine group—holds a prominent position. Lidocaine is included in the WHO’s list of essential drugs because of its effectiveness, safety, and cost efficiency across healthcare systems. Meta-analyses have consistently highlighted the benefits of perioperative lidocaine use, including pain reduction, shortened duration of ileus, decreased opioid requirements, and reduced hospital length of stay [8–11]. In addition to its application in surgical settings, lidocaine has been demonstrated to be useful in various medical contexts. For instance, Groudyne et al. [12] reported a 50% reduction in opioid consumption during genitourinary procedures, whereas in obstetric-gynecologic settings, lidocaine administration was associated with minimal hemodynamic changes and had no significant effect on Apgar scores [13]. However, despite the extensive evidence supporting the perioperative use of lidocaine, its specific application in obese patients undergoing bariatric surgery remains relatively underexplored. Therefore, the aim of this study was to assess postoperative pain outcomes following perioperative lidocaine infusion in this patient population.

## Material and methods

### Study design

We conducted a retrospective analysis of prospectively collected data from all patients aged over 18 years with a BMI exceeding 30 kg/m^2^ who underwent laparoscopic bariatric surgery (including bypass, gastric sleeve, and conversion procedures) at Centro Médico ABC in México City between January 1, 2021, and September 1, 2022. Patients who were allergic to local anesthetics and those with a BMI lower than 30 kg/m^2^ were excluded. Approval was obtained from the Ethics and Research Committee of our institute (code TABC-23–124).

### Study groups

Two study arms were established: patients who received an infusion of lidocaine and a control group. Patients in the control group received lidocaine solely as a bolus during induction, which was calculated at 1 mg/kg of the patient’s actual body weight. A structured database was constructed, including anthropometric data (sex, age, BMI, weight, height), preexisting comorbidities, history of substance abuse, drug usage, history of COVID-19, and prior surgeries. The anesthetic data included airway predictors, drugs administered during induction, laryngoscopy specifics (such as the type and number of blades used, Cormack–Lehane classification), endotracheal tube details, total lidocaine and fentanyl doses infused, and total doses of adjunctive medications administered, such as clonidine, dexamethasone, and ondansetron. Perioperative and postoperative complications such as nausea, vomiting, and pain were recorded. Surgical procedure details, including the type of surgery, total operative time, and length of hospital stay, were also documented.

### Lidocaine protocol

In the lidocaine infusion group, patients received a bolus dose of 1 mg/kg on the basis of actual body weight during induction, followed by a continuous infusion of 40 mcg/kg/min [14] during the first hour of the procedure. The infusion rate was subsequently adjusted to 20 mcg/kg/min for the remainder of the surgery, with both doses calculated on the basis of the predicted weight. Lidocaine infusion was discontinued 5 min before the surgical closure was completed.

### Perioperative management

The anesthesia machine and resuscitation equipment were checked before each case. Every patient was monitored via electrocardiography, capnography, pulse oximetry, and intermittent noninvasive blood pressure. Certified anesthesiologists conducted all procedures. Preoxygenation via a face mask was performed while the participants were in the ramped position for 2 min. Intravenously, midazolam (0.3 mg/kg), fentanyl (3–5 mcg/kg), a bolus of lidocaine (1.0 mg/kg), propofol (2 mg/kg), and cisatracurium (150–200 mcg/kg) were administered. Laryngoscopy was performed with a 3.0/3.5/4 mac blade, followed by endotracheal intubation with a 7.0/7.5/8.0 tube, confirmed by capnography, auscultation, and inspection. Protective mechanical ventilation was administered after intubation, and general anesthesia was maintained with desflurane inhalation at 1.0 °C (5–7% volume). Standard doses of dexamethasone (8 mg) and ondansetron (8 mg) were also administered perioperatively. The perioperative management protocol established the administration of clonidine by infusion at 0.2 mcg/kg/min, calculated on the predicted body weight. Clonidine was administered as part of the bariatric surgery group protocol, serving as an adjunct for analgesia, alongside fentanyl infusion. From a surgical point of view, all patients underwent laparoscopic gastric bypass with five ports. No abdominal cavity drainage, nasoenteric tube, or bladder tube was utilized. Postoperative analgesia included fentanyl infusion (1–1.5 mcg/kg/h) administered up to 30 min before the end of the surgical procedure. All patients were monitored postoperatively in the post-anesthesia recovery room before transfer to the ward. Postoperative analgesic regime was planned and administered by the physician in charge of the ward.

### Outcomes

Our primary outcome focused on assessing the presence of postsurgical pain following lidocaine infusion, which was measured via the Numerical Rating Scale (NRS) for pain intensity. Pain levels were evaluated at three time points: immediately after the procedure, prior to discharge from the postsurgical recovery room, and 24 h post-surgery. Pain intensity was categorized as mild (scores of 1–3), moderate (scores of 4–7), or intense (scores of 8–10) on the basis of the NRS scoring system.

The secondary outcome involved monitoring total opioid consumption until discharge from the post-surgical recovery unit, which was documented by recording the total number of analgesic rescues required post-surgery. Additionally, the presence of nausea and vomiting was assessed through direct questioning of the patients. These secondary outcomes provided insight into the overall analgesic requirements and incidence of common postoperative complications such as nausea and vomiting in the study population.

### Statistical analysis

We initially assessed the normal distribution of the data via the Shapiro–Wilk test. Quantitative variables are presented as medians with interquartile ranges, whereas qualitative variables are expressed as frequencies with percentages. Comparisons between groups (lidocaine perfusion vs. no lidocaine perfusion) were conducted via the Mann–Whitney *U* test for quantitative data and the chi–square test or Fisher’s exact test for qualitative data. For the primary objective, various generalized linear models were employed, whereas for the secondary objective, a generalized linear model with a binomial function was utilized. The performance of these models was evaluated via the adjusted *R*^2^ statistic and the Akaike information criterion (AIC). All the statistical analyses were performed via the SPSS v20 software for Mac.

## Results

**Fig. 1:**
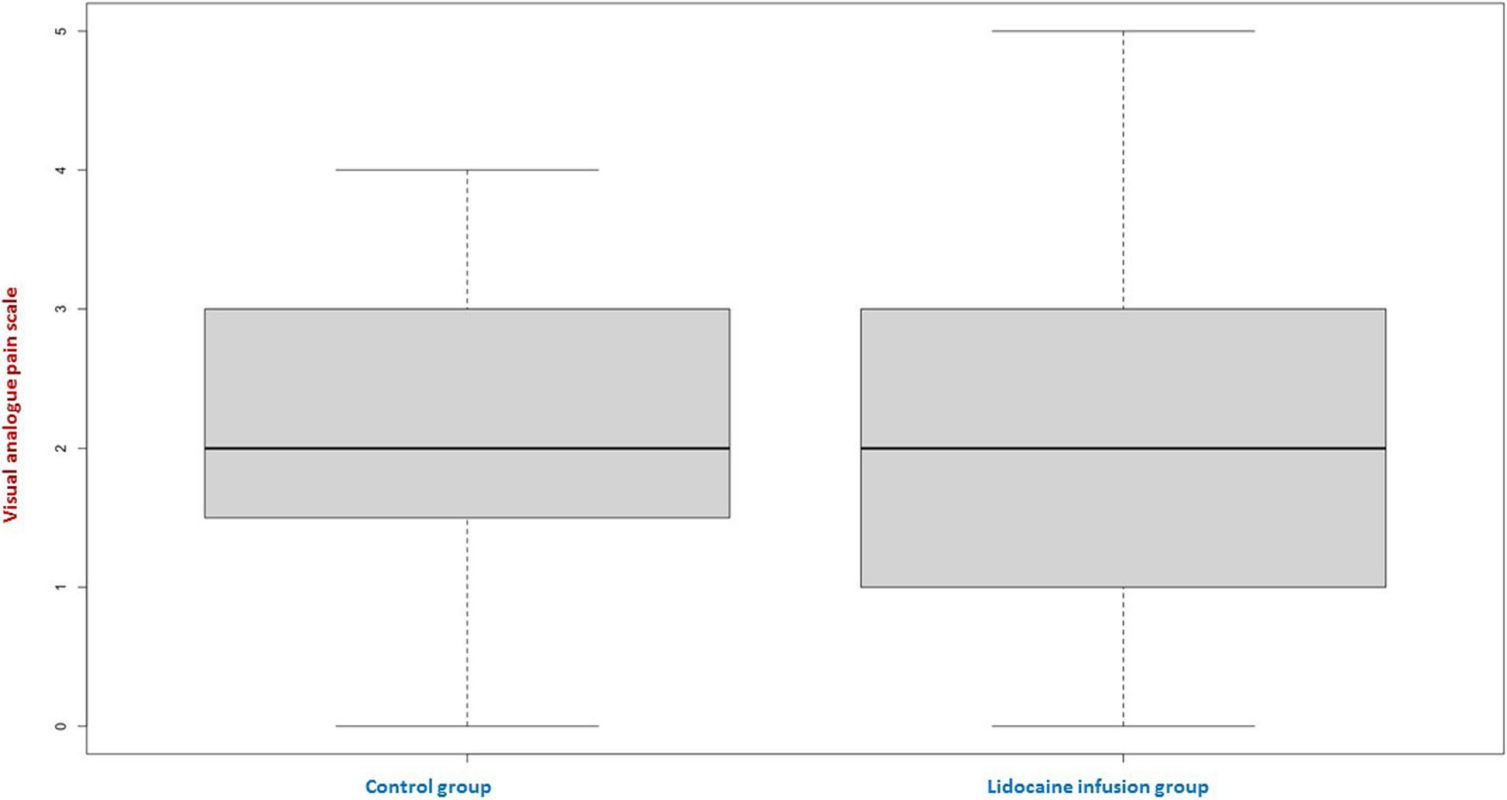
Box-and-whisker plot comparing both study groups (lidocaine infusion vs. no lidocaine infusion) in relation to pain intensity measured by the visual analog scale (VAS) at 24 h after surgery

**Fig. 2:**
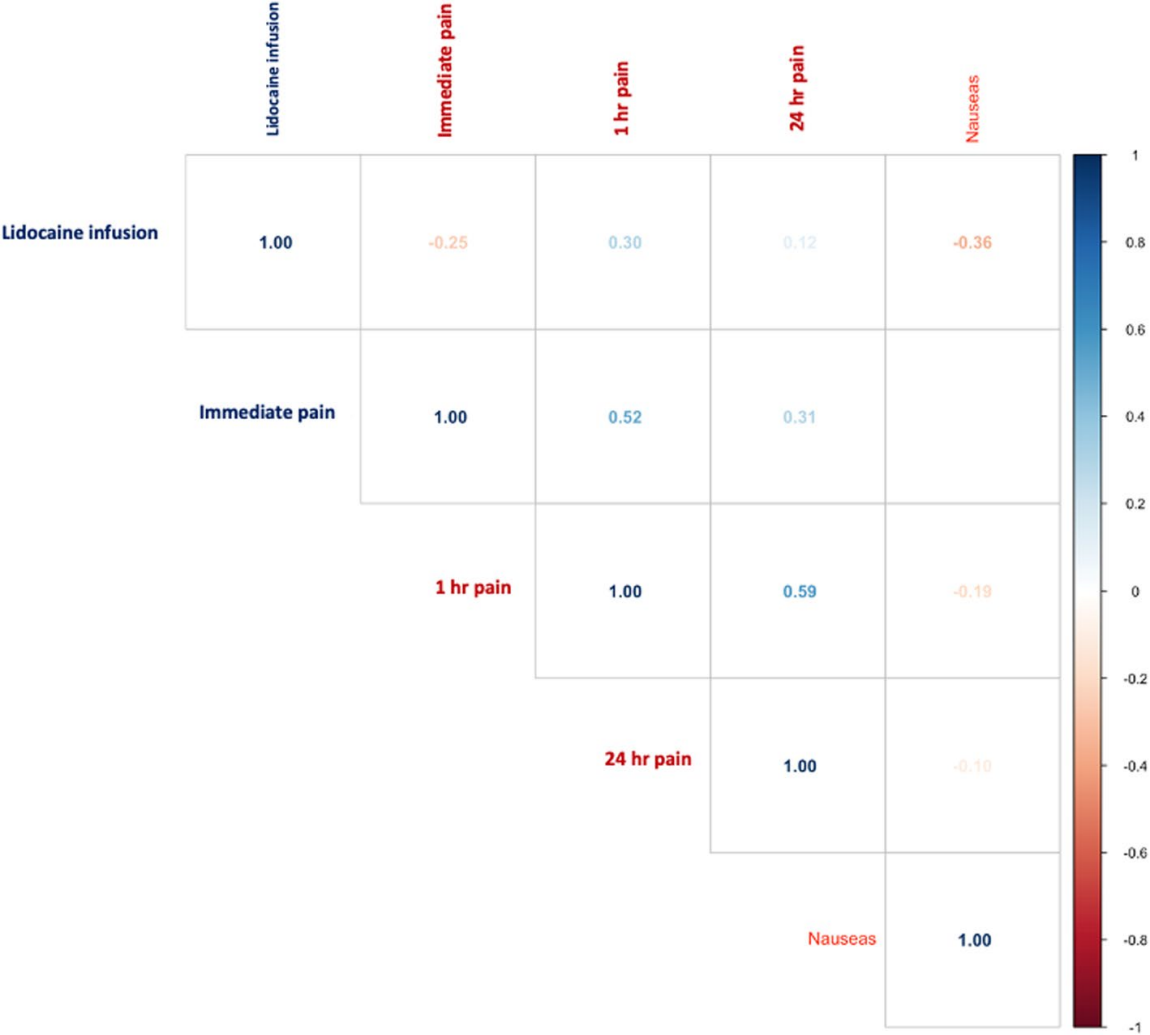
Spearman correlation between the study variables showing the rho coefficient for the different outcome measured

A total of 50 patients who underwent bariatric surgery were included; 29 (58%) were female, the average age was 39 years (ranging from 23 to 54), and the average body mass index (BMI) was 38.9 (ranging from 30.49 to 59.18). The remaining anthropometric characteristics are presented in Table 1. Among the total sample, 22 patients (44%) were in the lidocaine perfusion group, whereas 28 (46%) were in the control group. The intensity of pain at 24 h did not significantly differ between the two groups (*p* = 0.65), as illustrated in Fig. 1. The generalized linear model with an identity function between lidocaine infusion and pain at 24 h did not yield a significant difference (*p* = 0.42; 95% CI − 0.42–1.02). Furthermore, a significant positive correlation was observed between lidocaine infusion and pain at 1 h (rho = 0.30, *p* = 0.04). No statistically significant differences were observed between the two groups upon arrival at the post-anesthesia care unit (PACU) or during the first postoperative hour. When the two groups were compared, significant differences were observed in the total lidocaine administered (*p* < 0.001) and total clonidine administered (*p* = 0.009), both of which were greater in the lidocaine infusion group. Conversely, the group without lidocaine perfusion presented more cases of nausea (*p* = 0.01). Other variables compared between the groups are presented in Table 2. Additionally, there was a negative correlation between lidocaine and nausea (rho = -0.36, *p* = 0.003), as depicted in Fig. 2.

**Table 1:** Anthropometric data and surgical characteristics

	*N* = 50 (100%)
Lidocaine group^a^	22 (44)
Sex, male^a^	21 (42)
Age (years)^b^	39 [23–54]
Weight (kg)^b^	106.7 [85 -187]
Height (m)^b^	1.65 [1.54–1.82]
BMI (kg/m^2^)^b^	38.8 [30.49–59.8]
**Surgery**^**a**^	
Bypass	47 (94)
Gastric sleeve	2 (4)
Conversion	1 (2)
Surgery time (min)^b^	149 [80–185]
Surgery complications^a^	3 (6)
Bleeding from surgical staples^a^	1 (2)
Melena^a^	2 (4)
Nauseas^a^	19 (44)

^a^Frequency (percentage)

^b^Median [interquartile range]

**Table 2:** Results of the comparison between groups

	**Lidocaine infusion**	**Control**	***p* value**
**Sex, male**^**a**^	7 (31.8)	14 (50)	0.19
**Age (years)**^**b**^	39 [33–54]	40 [23–48]	0.86
**BMI (kg/m**^**2**^**)**^**b**^	38.6 [35.8–59.8]	35.9 [30.5–42.4]	0.30
**Lidocaine total (mg)**^**b**^	317.5 [272–381]	100 [80–100]	< 0.0001
**Fentanyl**^**b**^	450 [437.5–500]	500 [450–500]	0.09
**Pain**^**b**^			
** Immediate**	2 [0–3]	2 [2–4]	0.06
** 1 h post-surgery**	3 [1–4]	2 [0–2]	0.04
** 24 h post-surgery**	2 [1–3]	2 [1–3]	0.65
**Nauseas**^**a,b**^	4 (18.1)	15 (53.6)	0.01

^a^Frequency (percentage)

^b^Median [interquartile range]

## Discussion

Our study investigated the efficacy of perioperative intravenous lidocaine infusion for postsurgical pain management in bariatric surgery patients. The key findings of our analysis revealed no significant difference in pain intensity at 24 h post-surgery between the lidocaine infusion group and the control group (*p* = 0.65). Interestingly, we observed a significant positive correlation between lidocaine infusion and pain at 1 h post-surgery (rho = 0.30, *p* = 0.04). However, a notable finding was the negative correlation between lidocaine infusion and the incidence of postoperative nausea (rho =  − 0.36, *p* = 0.003), suggesting a potential benefit in reducing this common postoperative complication. The proposed mechanism of action for lidocaine in preventing nausea and vomiting may be related to enhanced gastrointestinal recovery or an opioid-sparing effect. Additionally, our results did not show significant differences in opioid consumption between the two groups (*p* = 0.09).

The implementation of ERAS protocols across various surgical disciplines has provided comprehensive guidelines for the multidisciplinary management of patients throughout the perioperative period [15, 16]. Bariatric surgery, as a relatively novel therapeutic approach, necessitates a meticulous anesthetic and surgical strategy due to the vulnerability of the patient population. Among the emerging therapeutic modalities, multimodal analgesia has gained prominence, with various adjunctive medications, such as lidocaine, magnesium sulfate, alpha agonists, and ketamine, being utilized to optimize pain management. Lidocaine has demonstrated a broad spectrum of safety and efficacy in this context and has been extensively studied. For example, De Oliveira et al. [17] conducted a randomized clinical trial demonstrating that perioperative lidocaine infusion (2 mg/kg/h) in bariatric procedures improved recovery and reduced opioid consumption. Conversely, findings from Tovikkai et al. [18] with a cohort of 345 patients undergoing laparoscopic bypass surgery suggested no decrease in opioid consumption following lidocaine infusion (0.5–5 mg/kg/h) at 24 h post-surgery. Similarly, Plass et al. [19], with a smaller sample size of 178 patients, did not observe significant differences in oxycodone consumption after surgery with a lidocaine infusion titrated at 1 mg/kg/h. Consistent with these studies, our data did not reveal significant differences in opioid consumption between the two groups of patients (*p* = 0.09). In addition to evaluating opioid consumption, our study investigated secondary objectives, such as the incidence of nausea and vomiting, which have been less explored in the bariatric population. Studies by Sakata et al. [20] and Plass et al. [19] did not find significant differences in the incidence of nausea and vomiting following lidocaine infusion (2 mg/kg/h and 1 mg/kg/h, respectively). However, our findings suggest a positive correlation between perioperative lidocaine infusion and the presence of nausea, albeit with a lower incidence (*p* = 0.01). Currently, a major trend in postoperative pain management is the use of regional anesthesia through ultrasound-guided blocks. As described by De Cassai et al. in a meta-analysis, the transversus abdominis plane (TAP) block has been shown to be superior to other regional anesthesia techniques in reducing opioid consumption, postoperative pain, nausea, vomiting, and the need for rescue analgesics in bariatric surgery [21]. Similarly, Nair et al. report that the application of the erector spinae plane block (ESPB) provides opioid-sparing analgesia and improved pain scores compared to control groups [22]. Both authors offer valuable tools for a more tailored and effective approach to postoperative pain management in bariatric surgeries. This retrospective study on the efficacy of perioperative intravenous lidocaine infusion for postsurgical pain management in bariatric surgery patients has several strengths and limitations.

One of the main strengths of this study is the homogeneous study population, which helps minimize confounding factors and enables more reliable comparisons between the lidocaine infusion and control groups. The study also addresses secondary objectives related to postoperative nausea and vomiting, which have been less explored in the bariatric population, providing valuable insights into these common complications. Furthermore, the sample size of 50 patients is comparable to that in previous reports in the field, adding to the validity of the findings. However, the study’s retrospective design is a limitation, as it may introduce bias and hinder the establishment of causal relationships. The relatively small sample size, although comparable to that of other studies, may limit the generalizability of the results to larger populations. Additionally, the study does not provide information on the long-term effects of lidocaine infusion on pain management and patient outcomes, as it focuses on the immediate postoperative period. Despite these limitations, this study offers valuable insights into the potential benefits of lidocaine infusion in mitigating postoperative nausea in bariatric surgery patients, suggesting its role as a component of multimodal anesthesia strategies. Further research with larger, prospective studies is needed to confirm these findings and explore optimal dosing and administration protocols for lidocaine infusion in the context of bariatric surgery.

## Conclusions

Intraoperative lidocaine infusion may have limited efficacy in managing postoperative pain in bariatric surgery patients, but it results in a notable improvement in the presence of postoperative nausea.

## Data Availability

The datasets used and/or analyzed during the current study are available from the corresponding author upon reasonable request.
